# A Case of Bullous Pemphigoid: A Prevalent and Potentially Fatal Condition

**DOI:** 10.7759/cureus.2533

**Published:** 2018-04-25

**Authors:** Jorge Parellada, Yanetsy Olivera Arencibia, Harold Watson, Nicole Parellada, Lara E Saikaly, Sami K Saikaly

**Affiliations:** 1 Internal Medicine, Orlando Regional Medical Center; 2 School of Medicine, Geisinger Commonwealth School of Medicine; 3 Department of Psychology, University of Florida, Gainsville; 4 College of Medicine, University of Central Florida College of Medicine

**Keywords:** bullous disease, bullous pemphigoid, treatment, dermatology, autoimmune

## Abstract

Bullous pemphigoid is the most common of the blistering disorders. It is most commonly found in the elderly and is diagnosed based on clinical, histologic, and immunologic criteria. It presents clinically with diffuse eczematous, pruritic, urticaria-like lesions, with the later appearance of tense bullae or blistering lesions typically filled with clear fluid. Histologically, a sub-epidermal blister is seen and immunofluorescence shows immunoglobulin G antibodies directed against the structural components of the keratinocytic hemidesmosomal proteins BP180 and BP230. Multiple treatment modalities are present for this condition, including anti-inflammatory medications, medications that reduce antibody formation, and treatments to increase the elimination of antibodies. The aim of this case report is to present a classic case of this condition, to highlight an awareness of differing treatment options, and to advocate referral to a dermatologist given its potential severity.

## Introduction

Bullous pemphigoid (BP) is the most common of the blistering disorders [1]. It is most commonly found in the elderly [1-2] and is diagnosed based on clinical, histologic, and immunologic criteria [2-4]. Multiple treatment modalities are present for this condition, including anti-inflammatory medications, medications that reduce antibody formation, and treatments to increase the elimination of antibodies [2,4]. The aim of this case report is to present a classic case of this condition, to highlight an awareness of differing treatment options, and to advocate referral to a dermatologist given its potential severity.

## Case presentation

**Figure 1:**
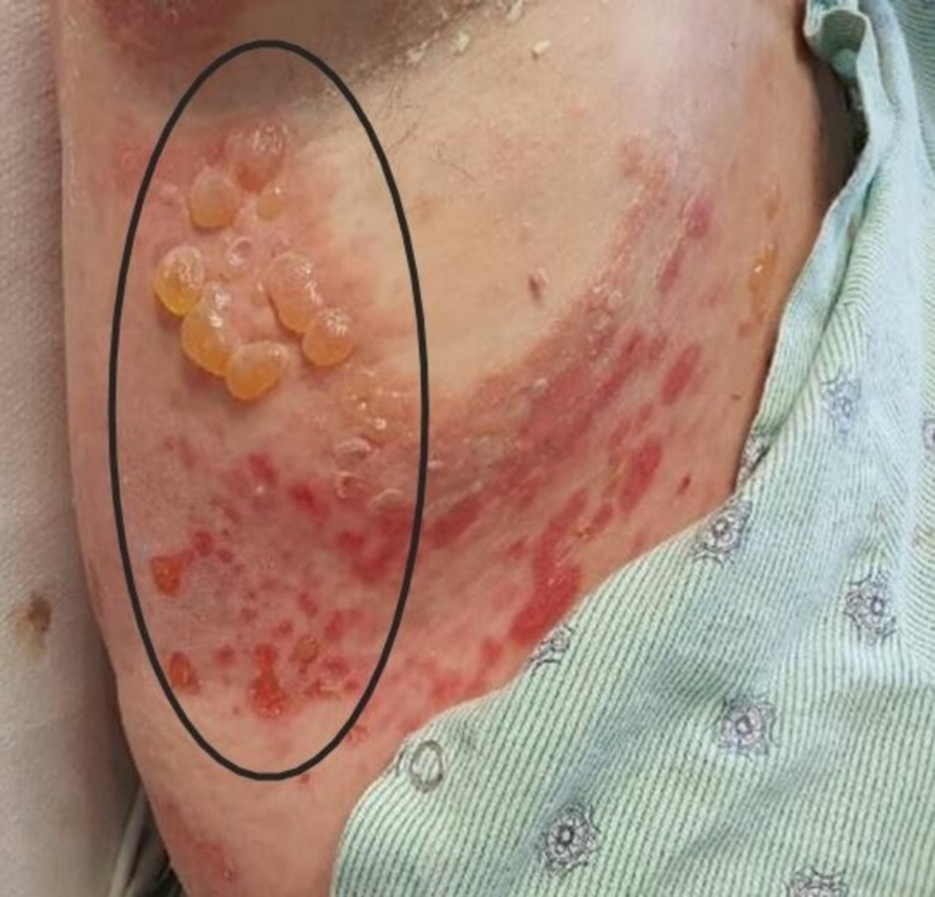
Clinical Lesions Diffuse and erythematous tense bullae

A 57-year-old Caucasian female presented to the hospital with a worsening, diffuse, bullous eruption. The eruption started four weeks prior and was distributed mainly on her lower extremities. The patient went to her primary care physician, who prescribed doxycycline and sulfamethoxazole/trimethoprim and told the patient that she had cellulitis. The patient took the antibiotics but the rash continued to worsen. After completing the antibiotic course without improvement, the patient presented with diffuse and erythematous tense bullae ranging from 1.5 to 2 centimeters in diameter. The lesions can be appreciated on the patient’s face, neck, back, chest, abdomen, and extremities (Figure 1).

Some of the lesions had ruptured and were both pruritic and painful.

**Figure 2:**
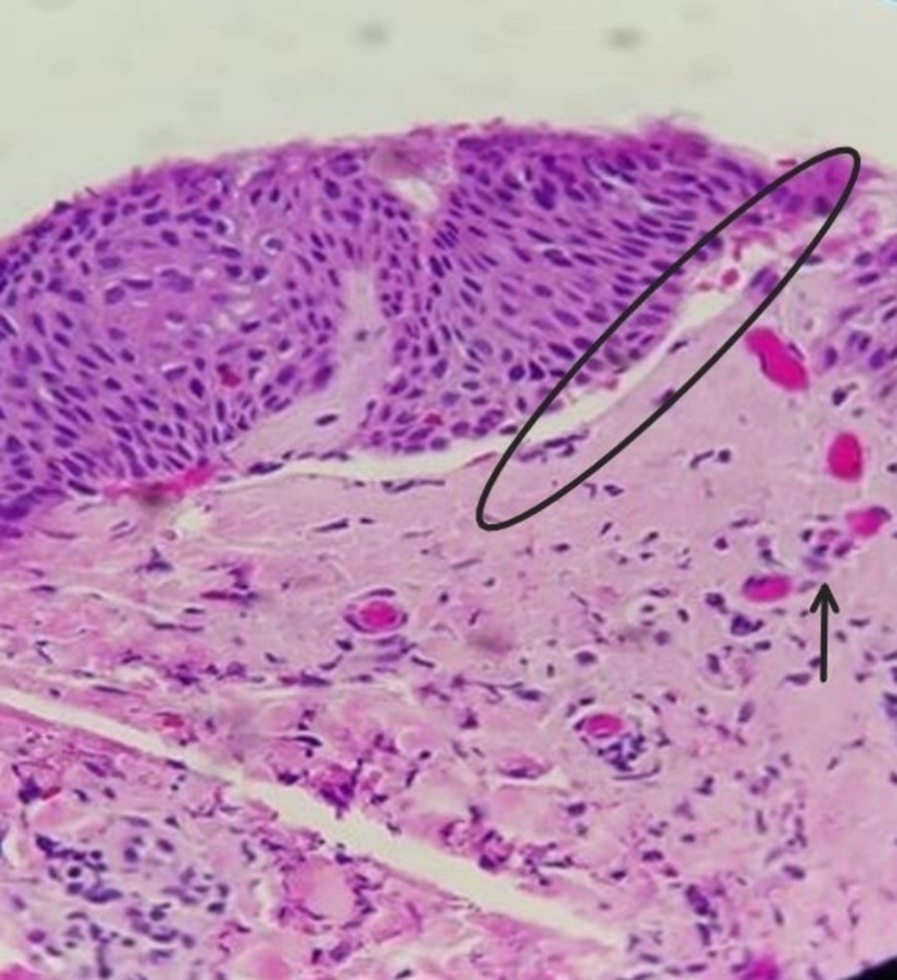
Histology Subepidermal blister (oval) with eosinophils and neutrophils and underlying dermis demonstrating severe edema and infiltrate composed of eosinophils and lymphocytes (arrow)

The patient was afebrile and without leukocytosis, yet C-reactive protein was elevated at 97.8 mg/L. An initial punch biopsy was performed and returned negative for a definitive diagnosis. A repeat punch biopsy four days later showed a subepidermal blister with eosinophils and neutrophils. The underlying dermis demonstrated severe edema and infiltrate composed of eosinophils and lymphocytes (Figure 2).

**Figure 3:**
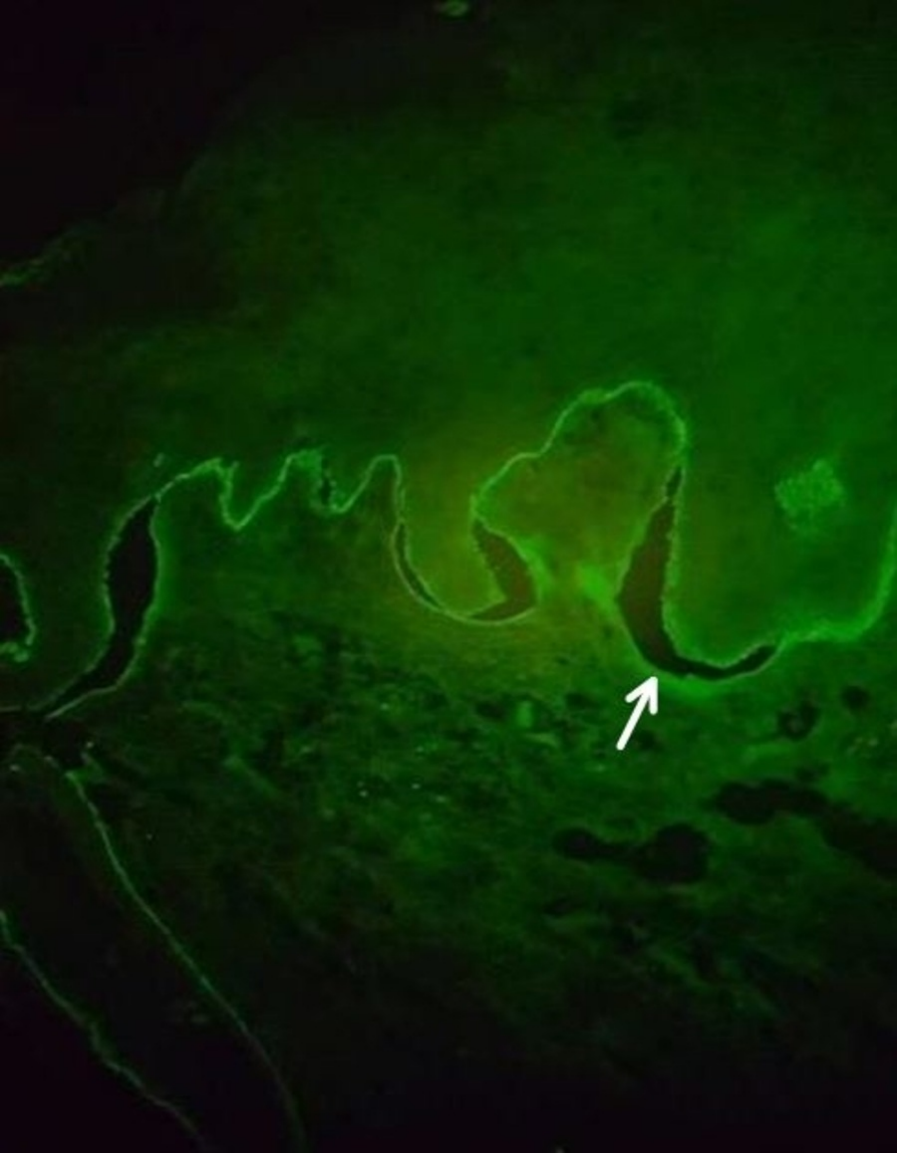
Immunofluorescence Linear deposition of immunoglobulin G and complement C3 along the dermo-epidermal junction (white arrow)

Direct immunofluorescence (DIF) of the skin revealed the linear deposition of immunoglobulin G (IgG) and complement C3 along the dermo-epidermal junction (Figure 3).

The patient was diagnosed with bullous pemphigoid and was treated with prednisone 60 mg daily. The patient responded well with a decreased number of bullae, as well as an improvement of the erythema and pruritus. The patient was discharged on a tapering regimen of prednisone 60-40-20 mg for one month per dose, along with oral sulfamethoxazole/trimethoprim 160 mg daily for prophylaxis of Pneumocystis carinii pneumonia. The patient was referred to a dermatologist to discuss adding a steroid-sparing agent such as methotrexate or azathioprine.

## Discussion

Pemphigoid is derived from the Greek words pemphix (bulla, blister) and eidos (form). BP is the most common of the blistering disorders, with an incidence of 0.2 to three cases per 100,000 persons each year [1]. The disease does not have a gender bias but is most commonly found in individuals above 75 years of age [1-2]. Young adults and children may also be affected, but much less frequently.

BP is an autoimmune disorder characterized by the presence of IgG antibodies directed against the structural components of the keratinocytic hemidesmosomal proteins BP180 and BP230 [1-3]. Both the antibody and complement components deposit along the basement membrane at the level of the dermo-epidermal junction, triggering a destructive inflammatory response with the formation of the characteristic blistering lesions [1-3]. Antibody levels will directly correlate with disease activity [2-3]. Focal separation of the epidermis and dermis results in tense subepidermal blister formation.

BP is characterized initially by the development of diffuse eczematous, pruritic, urticaria-like lesions, with the later appearance of tense bullae or blistering lesions typically filled with clear fluid. Lesions are located mainly in the trunk, flexor compartments of the extremities, and the axillary area [3]. The blisters usually heal without scarring, and their appearance follows a waxing and waning course [2-3].

Atypical forms of BP can occur [2]. These can present as a localized pemphigoid that can be seen mainly in the lower legs, genital area, and sites of trauma. This can progress to a more generalized form. Other presentations include dyshidrosiform pemphigoid that affects the palms and soles, erythrodermic pemphigoid, nodular pemphigoid, and lichen planus pemphigoid. Mucosal involvement is not frequent and when it occurs, it is usually limited to the mouth.

The clinical characteristics of BP may resemble a variety of other skin conditions. It is important to use clinical, histologic, and immunologic direct immunofluorescence (DIF) findings to differentiate BP from other blistering disorders like dermatitis herpetiform, epidermolysis bullosa acquisita, pemphigus vulgaris, etc. [2]. This is important, as misdiagnosis can lead to mistreatment and, eventually, worse outcomes for patients with a potentially treatable condition.

BP is diagnosed based on clinical, histologic, and immunologic criteria. A group of French authors proposed the following clinical predictors of BP: absence of atrophic scars, limited neck or head involvement, absence of mucosal involvement, and age greater than 70 years. The presence of three out of these four criteria for BP had a sensitivity of 90%, a specificity of 83%, and a positive predictive value of 95% [5].

To confirm the diagnosis via histology, two punch biopsies should be taken. One skin biopsy should be from the edge of an intact blister fixed on formaldehyde for hematoxylin and eosin (H&E) staining. Typical findings include a subepidermal blister with a perivascular inflammatory infiltrate and eosinophils. Eosinophilic spongiosis is a characteristic feature and highly suggestive of BP [2]. A second skin biopsy from normal perilesional tissue should be taken for DIF and placed in saline or Michel’s solution media. The characteristic DIF findings are linear IgG and C3 deposits along the basement membrane [2]. DIF is considered the gold standard for diagnosis.

Several immunologic tests exist to determine the antigenic specificity of antibodies. Enzyme-linked immunosorbent assay (ELISA) for bullous pemphigoid antibodies is available. These detect IgG antibodies that react with BP180 (the most common antigenic target) and with BP230 [2,4]. The levels of anti-BP180 have been found to directly correlate with disease activity [2-3]. While these tests are available, they may not be used routinely for clinical diagnosis if clinical presentation and biopsy are sufficient.

BP is a potentially fatal disease, with a recent French study of 502 patients reporting a one-year mortality of 38 percent [6]. A Swiss study of 115 patients with BP found probabilities of death one, two, and three years following diagnosis to be 21, 28, and 39 percent, respectively. The mortality rate was three times higher for individuals diagnosed with BP than for age and sex-matched subjects in the general population [7]. Given these findings, the correct diagnosis and treatment of BP are crucial to improve the outcomes in this patient population.

Three categories of drugs may be used to treat BP [2,4]. The first category is anti-inflammatory drugs, such as topical steroids, sulfonamides, and antibiotics with anti-inflammatory properties like tetracycline. Another drug class consists of those that decrease the production of antibodies such as systemic steroids, azathioprine, methotrexate, mycophenolate, cyclosporin, and rituximab. Finally, treatments that increase the elimination of abnormal antibodies like plasmapheresis and intravenous immunoglobulin (IVIG) can be performed.

## Conclusions

Overall, BP is a potentially fatal disease with a high prevalence, which is treatable when under the appropriate level of care. This case report aims to increase disease awareness and highlight differing treatments, as well as to advocate referral to a dermatologist, given its potential severity.
